# The impact of a hands-on arthrocentesis workshop in undergraduate medical education

**DOI:** 10.1186/s12909-020-02174-6

**Published:** 2020-08-10

**Authors:** Andreas Ladurner, Thomas Nijman, Tiffany K. Gill, Peter J. Smitham

**Affiliations:** 1 Department of Orthopaedics and Trauma, Royal Adelaide Hospital, Port Road, Adelaide, SA 5000 Australia; 2 Centre for Orthopaedic and Trauma Research, The University of Adelaide, Adelaide, Australia; 3 Adelaide Medical School, Adelaide, SA Australia

**Keywords:** Arthrocentesis workshop, Synthetic joint model, Medical student’s education, Skills enhancement

## Abstract

**Background:**

To evaluate the impact of a training programme for arthrocentesis on procedural skills enhancement and self-confidence in medical students.

**Methods:**

Participants were provided a structured workshop on injection models. A self-confidence questionnaire and medical knowledge assessment were performed. Retention of knowledge and skills were assessed at a later time point during a formal OSCE examination and compared to participants who had not attended a lecture and clinical attachments only. *P*-values, 95% confidence intervals about the mean, standard error of the mean, and standard deviations of the differences were calculated.

**Results:**

All participants gained self-confidence, and improvement of their skills was significant. The mean self-confidence with performing an arthrocentesis procedure increased from 1.3 pre- to 5.9 points post-workshop (10-point Likert scale). The knee was the joint students felt most confident with (1.3 to 6.5 points). Knowledge on the selection of corticosteroid preparations (1.2 to 5.8 points) gained substantially, as well as confidence in providing post-injection advice (1.9 to 6.6 points). Upon the OSCE examination, attendance to the workshop resulted in a significant higher total score (16.2 vs 14.8 points, *p* < 0.05).

**Conclusions:**

A workshop for arthrocentesis procedures, in conjunction with other learning activities, is well suited to increasing skills and self-confidence in fourth year medical students and allows for developing important baseline knowledge and practicing invasive techniques without risk to a patient.

**Trial registration:**

This trial has been approved by the human research ethics committee of the University of Adelaide (Ethics approval No H-2019-134).

## Background

Disorders of the musculoskeletal system are estimated to cause one in four consultations in primary care according to the World Health Organisation, ranking musculoskeletal problems first among the most common reasons to seek medical care [1]. Pain and disability are common symptoms, which not only impact on the individual’s quality of life, but also on people’s ability to earn a living and be independent. Up to 60% of all disability pensions are thought to be related to musculoskeletal conditions [1]. Primary care physicians, however, report low confidence in performing musculoskeletal examinations and arthrocentesis procedures, despite the frequency of musculoskeletal complaints encountered in ambulatory clinics [2]. Up to 95% of primary care providers who practice aspirations report that they have been inadequately trained in joint injection techniques during undergraduate and postgraduate training [2, 3]. Surveys show that undergraduate medical students spend very little time on the musculoskeletal system, both in basic science and in clinical training [4].

Hence, international efforts have been focused on improving medical student musculoskeletal basis skills to adequately address the need [5], and a swift implementation is sought through undergraduate curricular reform. The literature supports the use of different instructional methods that engage learners and provide meaningful learning contexts [6, 7]. As for arthrocentesis procedures, didactic lectures are useful to provide basic information to medical students [7, 8], but lack the opportunity to practice and thereby integrate knowledge into skills. The use of synthetic models in teaching arthrocentesis has been shown to result in long-lasting improvements in the skill and confidence with procedures among undergraduate and postgraduate medical personal [9, 10]. The benefit of synthetic anatomic models are that they are (relatively) low cost, portable, are easily accessible, and reusable [11]. There are a number of different synthetic models available on the market with some allowing students to aspirate fluid whilst others provide an electronic feedback in the form of a light if the correct area is touched by the needle.

As clinical opportunities to perform arthrocentesis are limited, a half-day arthrocentesis workshop for fourth year medical students was developed with the aim of introducing the basic concepts of arthrocentesis and increasing self-confidence in the performance of these procedures along with an increased understanding of the indications, injection options and possible complications. The objective of this study was to evaluate the impact of the hands-on workshop for arthrocentesis procedures on procedural skills enhancement and self-confidence in fourth year medical students.

## Methods

### Participants

The bachelor of medicine / bachelor of surgery (MBBS) course within the University is a six year course. The majority of clinical teaching begins in the 4th year with approximately 150 students per year. After graduation students will enter a specialty-specific intern period during their year with some undertaking a musculoskeletal (MSK) or emergency medicine internship for 3 months. The arthrocentesis workshop was held for medical students during their 4th year of training. As the program was introduced during a running year, 15 students missed the opportunity to participate, because the program was only introduced after those students had absolved their musculoskeletal module. After its implementation, the program was offered as a non-compulsory training opportunity for the students absolving the musculoskeletal module. There was no formal recruitment process. Apart from the workshop, all students had access to online learning material, a one-hour lecture on dislocation management and aspirations and injections as well as access and training to the injection models for 20 min per model over 3 weeks as part of their weekly anatomy sessions.

### Workshop design

The workshop was designed as a half-day program. The goal of the program was to introduce the basic concepts of arthrocentesis and increase self-confidence in the performance of these procedures. Trainees received both didactic and hands-on instruction. There was a one-hour lecture, during which general principles related to the arthrocentesis procedure were outlined. The students were familiarized with indications and contraindications to the procedure, and the equipment required for safe performance of an arthrocentesis. Topics such as the consent process, risks of corticosteroid injection and site-specific anatomy including approaches were evenly analyzed. The models available and the approaches for arthrocentesis discussed are shown in Table 1. The lecture was followed by a hands-on workshop. Arthrocentesis was demonstrated by a faculty member and was performed for each injection model. The students were then allowed to practice their technique on the different models under supervision until they felt comfortable with the procedure for each of the anatomical sites. Written guidelines outlining the important steps of the arthrocentesis procedure were also available at each work station (Additional file 1 shows the guidelines for the knee station).

**Table 1:** Models and approaches

Knee	- Superolateral - Superomedial - Inferior
Shoulder	- Posterior approach to the glenohumeral joint - Lateral approach to the subacromial space - Superior approach to the acromioclavicular (AC) joint
Elbow	- Lateral approach to the elbow joint - Direct approach to the medial epicondyle - Direct approach to the lateral epicondyle
Spine	- Lumbar punction

To ensure the workshop was meeting its objectives, a self-assessment questionnaire and medical knowledge test were administered before and after the session. The workshop finished with an impact survey that was completed optionally by the participants.

### Models

A total of four knee models, one shoulder, one elbow and two spine models were available. All models were “Limbs and Things” (Bristol, England). The models allowed for palpation of anatomic landmarks and featured skin like coverings that tolerated multiple needle sticks without showing former aspiration sites. The knee models were filled with artificial synovial fluid that could be aspirated upon proper positioning of the aspirating needle. The shoulder and elbow models featured electronic feedback indicating correct and incorrect positioning of the needle. The spine models allowed for fluid aspiration from the spinal canal to simulate a lumbar puncture.

### Self-assessment questionnaire

The questionnaire included 19 self-assessment questions and was distributed to all participants prior to the lecture and immediately after termination of the hands-on workshop (Additional file 2). The pre-workshop self-assessment also included 2 questions related to any previous experience with the procedure (“how many arthrocentesis of any site / of the knee have you performed by yourself prior to the workshop?”). The survey questions asked about the level of self-confidence in joint anatomy and performance of joint and soft tissue injections in general and in each of the anatomic sites being taught (knee, shoulder, elbow, spine). In addition, self-confidence with the indication of corticosteroids, quantity, frequency and choice of corticosteroids as well as confidence in providing post-injection advice was assessed. Responses were recorded on a 10-point Likert scale (0 = not confident at all, 9 = very confident).

### Medical knowledge check

The pre- and post-test sought evaluation of the knowledge about the anatomy of the injection sites that were discussed, indications and contraindications for arthrocentesis, corticosteroid preparations and side effects as well as synovial fluid analysis. It consisted of multiple choice and short answer questions (Additional file 3). The content of the test was subsequently covered during the didactic portion of the workshop. The post-workshop knowledge assessment was held immediately following the practice part.

### Postworkshop impact survey

An optional survey was also distributed to the participating students at the end of the workshop. Students were reassured that this survey was anonymized and would not affect their module score. They were told that the only purpose of the survey was to identify ways to improve the module. The survey evaluated the influence of the workshop on the skills and future habits of the participants as well as the impact on the confidence to perform an arthrocentesis on their own. Students were asked about the number of attempts needed before feeling confident in their injection skills. Finally, feedback on the quality of the models and the didactic instruction was solicited using a 10-point Likert scale (0 = poor, 9 = very good).

The questions of all surveys (self-assessment questionnaire, medical knowledge check and impact survey) were designed by the investigators to align with the established learning objectives of the MSK module. An internal validation of the questions was performed. Expert consent among the lecturers of the musculoskeletal module was used to achieve face/content validity.

### Skills assessment during OSCE examination

All 4th year medical students undergo a summative yearly skills assessment test (OSCE examination). Performance of a knee joint aspiration was among the possible scenario’s students could be assigned to during this evaluation. Thus, the aspiration scenario could be undertaken by students who had attended the workshop. Students assigned to the scenario were asked to perform an aspiration of the knee on a synthetic model after taking consent from the examiner. After successful arthrocentesis, laboratory results from a knee aspirate were shown to the examinee, and they was asked to report possible diagnoses and initial management options based on the findings. Each examinee was evaluated for professionalism (0 (min) to 6 (max) points), clinical skills (0 (min) to 9 (max) points) and reasoning (0 (min) to 5 (max) points) by a senior staff member of the University of Adelaide. The raters were blinded towards the participation status of the examinees. A total test score (0 (min) to 20 (max) points) was calculated summarizing all three subsections of the evaluation. The scores for those attending the workshop were compared to those who did not. This study was approved by the Ethics committee of the University of Adelaide (Ethics approval number H-2019-134).

### Statistical analyses

As the data were not normally distributed, a Wilcoxon signed rank test was used to evaluate whether or not there were significant changes in the scores given before and after the workshop. The null hypothesis was that the mean difference in ranks (corresponding to score shift) between the paired observations was zero, whereas the alternative hypothesis was that the mean difference between the paired observations was not equal to zero. Test significance was determined at the alpha level of 0.05. A positive mean difference indicated that the workshop was effective in increasing confidence and a negative mean difference indicated that the workshop lowered confidence. A mean difference near zero indicated that the workshop had little effect. *P*-values, 95% confidence intervals about the mean, standard error of the mean, and standard deviations of the differences were also calculated. All statistical analyses were performed with R (R: a language and environment for statistical computing. R Foundation for Statistical Computing, Vienna, Austria. URL http://www.R-project.org/).

## Results

### Personal experience

A total of sixty-nine students participated in the arthrocentesis workshop, while twenty-one (23%) students did not. Three students out of the 69 attending had performed one to five arthrocentesis procedures prior to the workshop, whilst the other 66 students did not have practical exposure towards the procedure up to that date.

### Self confidence

The pre-workshop confidence towards performing an arthrocentesis in general averaged 1.2 on the 10-point Likert scale. People were most confident performing an arthrocentesis of the acromioclavicular (AC) joint (1.5), as opposed to the glenohumeral joint, subacromial space or spine (1.4 each). The students were least confident performing an arthrocentesis of the knee or elbow (1.3 each). Confidence in the knowledge of the relevant anatomy was generally higher, reaching values between 4.4 (elbow) and 5.0 points (spine) respectively. Interestingly, students felt the highest confidence with knowledge of the anatomy of the spine but at the same time less confidence to perform an infiltration on it. In spite of being less confidence performing an arthrocentesis, participants overall felt quite comfortable to obtain consent for the procedure, with a mean of 5.5 points. Risks of arthrocentesis and indications for corticosteroids showed mean values of 3.9 and 4.0 in the 10-point Likert scale, whilst contraindications for corticosteroids scored 3.2 point on average. According to the answers provided, the students were not confident regarding the choice of the preparation, dosage or frequency of corticosteroid infiltrations as well as the provision of post-injection advice to patients. A detailed description of the self-assessment questionnaire results is given in Table 2.

**Table 2:** Self confidence level pre- and post-workshop

	Pre-Workshop (*N* = 69)	Post-workshop (*N* = 69)	Change		
	(mean, SD, min - max)	(mean, SD, min -max)	n	%	p
Anatomy					
- Knee	4.9 ± 1.7 (2–8)	6.2 ± 1.2 (5–8)	1.3	+ 24	< 0.001
- Shoulder	4.9 ± 1.6 (2–8)	5.9 ± 1.4(4–9)	1.0	+ 21	< 0.001
- Elbow	4.5 ± 1.6 (2–8)	5.8 ± 1.5(4–9)	1.3	+ 30	< 0.001
- Spine	5.0 ± 1.5 (3–8)	5.9 ± 1.6 (3–9)	0.9	+ 18	< 0.001
Performance of Arthrocentesis					
- In general	1.3 ± 1.7 (0–5)	5.9 ± 1.2 (5–9)	4.6	+ 369	< 0.001
- Knee joint	1.3 ± 1.8 (0–5)	6.5 ± 1.3(5–9)	5.2	+ 387	< 0.001
- Glenohumeral joint	1.5 ± 1.5 (0–5)	5.8 ± 1.5 (4–9)	4.3	+ 297	< 0.001
- Subacromial space	1.4 ± 1.5 (0–5)	5.3 ± 1.5 (4–8)	3.9	+ 270	< 0.001
- Acromioclavicular joint	1.5 ± 1 .8 (0–6)	5.3 ± 1.6 (2–8)	3.8	+ 258	< 0.001
- Elbow	1.3 ± 1.7 (0–5)	5.9 ± 1.4 (4–8)	4.6	+ 337	< 0.001
- Spine	1.4 ± 1.5 (0–5)	4.8 ± 1.4 (2–7)	3.4	+ 231	< 0.001
Obtaining consent for arthrocentesis	5.5 ± 2 (3–9)	6.9 ± 1.2 (5–9)	1.4	+ 26	< 0.001
Risks of arthrocentesis	3.9 ± 2.4 (0–8)	7.0 ± 1.3 (5–9)	3.1	+ 81	< 0.001
Indication for corticosteroids	4.1 ± 2 (0–8)	6.9 ± 1.2 (5–9)	2.8	+ 68	< 0.001
Contraindication for corticosteroids	3.2 ± 1.8 (0–7)	6.9 ± 1.2 (5–9)	3.7	+ 118	< 0.001
Choice of corticosteroid preparation	1.2 ± 1.3 (0–4)	5.8 ± 1.5 (3–8)	4.6	+ 382	< 0.001
Quantity of corticosteroid for injection	0.7 ± 1 (0–3)	6.0 ± 1.5 (3–8)	5.3	+ 763	< 0.001
Frequency of injection	1.4 ± 2.2 (0–8)	5.8 ± 2.2(0–8)	4.4	+ 303	< 0.001
Post-injection advice	1.9 ± 2 (0–7)	6.6 ± 1.4 (3–9)	4.7	+ 247	< 0.001

All values are provided as mean values of a 9-point Likert scale, (0 = not confident at all, 9 = very confident)

Overall, students gained significant benefit from the workshop in terms of confidence towards the procedure (*p* < 0.001 for all subsections of the questionnaire). The mean confidence levels range from 4.8 (performance of an arthrocentesis at the spine) to 6.9 points (obtaining consent for the procedure) on the 10-point Likert scale. The mean change in confidence when comparing each pre- and post-workshop answer showed values between 0.9 (+ 18%, anatomy of the spine) and 5.3 points (+ 763%, quantity of corticosteroid for injection). In general, the gain in self-confidence was less in the fields were the participants already felt quite comfortable prior to the workshop (anatomy, obtaining consent), whilst self-confidence increased substantially with performing an arthrocentesis procedure in general (+ 4.7 points / + 369%), knowledge on the selection (+ 4.6 points / + 382%), quantity (+ 5.3 points / + 763%) and frequency of corticosteroids in use (+ 4.4 points / + 303%) as well as providing post-injection advice (+ 4.7 points / + 247%). Students felt most confident in performing an aspiration of the knee (6.5 points), followed by the elbow (5.9 points) and glenohumeral joint (5.8 points). In contrast, confidence was less with procedures involving the spine (4.8 points).

### Medical knowledge assessment

All participants showed profound knowledge of the anatomy relevant to the arthrocentesis procedure. The students achieved a mean correct score of 17.9 (85.2%, range: 14 to 21 points, maximum score = 21) in the anatomical section of the pre-test. The result was even better in the post-workshop knowledge assessment, where the mean number of correct answers increased to 19.1 out of 21 (91.1%, range 14 to 21). This was an improvement of 7% (*p* < 0.001). All students correctly agreed on performance of a cell count and a synovial fluid culture as a diagnostic tool for synovial fluid analysis. Overall, 75.0% of the answers in the section on diagnostic synovial fluid analysis were completed correctly prior to the workshop, while 93.1% correct answers were obtained after the workshop (improvement 24%, *p* < 0.001). The lowest proportion of correct answers prior to the workshop were achieved with the questions to corticosteroid preparations (18.4% correct answers), followed by the question regarding incidence of infective complications after arthrocentesis (26.1% correct answers). Questions relating to corticosteroid preparations were answered correctly by 53.1% of students after the workshop (improvement 189%, *p* > 0.001), and 85.5% of all participants ticked the right answer when asked about the incidence of infection after arthrocentesis (improvement 228%, p > 0.001). Knowledge about contraindications and adverse effects of corticosteroid injections also improved after the workshop, but there was a high proportion of correct responses prior to the workshop. A summary of all of the answers is presented in Table 3.

**Table 3:** Medical knowledge check pre- and post-workshop

	Pre-Workshop (*N* = 69)		Post-Workshop (*N* = 69)		Change		
	n correct answers (mean, min – max))	%	n correct answers (mean, min – max))	%	N (mean)	%	p
Anatomy * (total number of correct answers: 21)	17.9 ± 0.5 (14–21)	85.4	19.1 ± 0.4 (14–21)	93.1	1.2	+ 7.0	< 0.001
Diagnostics / synovial fluid analysis ** (total number of correct answers: 9)	6.7 ± 0.5 (1–9)	75.0	8.4 ± 0.3 (4–9)	93.1	1.7	+ 24	< 0.001
Corticosteroid preparations *** (total number of correct answers: 3)	0.6 ± 0.2 (0–3)	18.4	1.6 ± 0.2 (0–3)	53.1	1	+ 189	< 0.001
Contraindications to intraarticular corticosteroid injections**** (total number of correct answers: 4)	2.9 ± 0.3 (0–4)	72.5	3.5 ± 0.2 (1–4)	88.4	0.6	+ 22	< 0.001
Adverse effects of intraarticular corticosteroid injections ***** (total number of correct answers: 4)	2.7 ± 0.3 (0–4)	68.1	3.7 ± 0.1 (2–4)	91.7	1	+ 35	< 0.001
Infection ****** (total number of correct answers: 1)	0.3 ± 0.1 (0–1)	26.1	0.9 ± 0.1 (0–1)	85.5	0.6	+ 228	< 0.001

*assign specific anatomic terms provided from a list to structures marked on anatomic drawings

** indicate on whether or not to perform specific laboratory studies for synovial fluid analysis, marking each given examination as “true” or “false”

*** select the most appropriate corticosteroid preparation (4 preparations provided) for each procedure provided

**** select 4 (relative) contraindications to giving intraarticular corticosteroid injections from a list (14 choices)

***** select 4 adverse effects of giving a single intraarticular corticosteroid injection from a list (14 choices)

****** tick the most appropriate answer to the rate of infection as a complication of arthrocentesis (6 choices)

### Impact survey

Overall, 68 out of 69 students voluntarily completed the impact survey and provided an overall positive feedback (Additional file 4). All students perceived an enhancement of their own skills with the arthrocentesis procedures, and all commented that the workshop would have changed their own future practice habits. Every participant would recommend the workshop to other students, and all favored similar workshops being mandatory for young doctors prior to performing arthrocentesis on their own. Nevertheless, only 80.9% of the students felt overall confident to perform unsupervised arthrocentesis procedures after the workshop with knee arthrocentesis the procedure students were most confident with (95.6%). On average, the students had 8.5 attempts before feeling comfortable with the procedure overall. The lowest confidence was recorded for infiltration procedures at the spine (44.4% confidence), followed by the AC joint (50% confidence). The results of the impact survey are presented in Table 4.

**Table 4:** Impact survey

	Positive answers (“yes”)	
	%	
Skills enhancement *	100	
Influence on future practice habits **	100	
Mandatory workshop before practicing ***	100	
Recommend the workshop ****	100	
Confident with the procedure *****:		
–		Attempts until
–		confident (n)
- Overall	80.9%	8.5 (1–22)
- Knee	95.6%	3.7 (1–20)
- Glenohumeral joint	72.1%	2.2 (1–5)
- Subacromial space	58.8%	1.8 (1–4)
- Acromioclavicular joint	50.0%	1.8 (1–4)
- Elbow	77.9%	2.6 (1–5)
- Spine	44.4%	2.5 (1–6)

*Do you think, this workshop is useful to enhance the skills in arthrocentesis technique

**Do you think, this workshop influenced your future practice habits?

***Do you think, every doctor should complete a workshop like this first before performing an injection on themselves?

****Would you recommend this workshop to other students?

*****Do you think you feel comfortable to perform an arthrocentesis on your own after completing this workshop? If so, how many attempts did it take until you felt comfortable with the procedure?

### Rating of the workshop and feedback

Overall, the workshop was rated 7.9 (4–9) on the 10-point Likert scale on average. The quality of the equipment provided scored 6.8 points on average (4–9), while the mean value for quality of the lecture was 8.1 points (5–9). Students were also given the opportunity to provide feedback as free text. The students praised the quality of the presentation and the didactic value of the course, but wished improvements to the models provided. Many students appreciated the possibility to aspirate fluid upon correct needle positioning as with the knee models, and wished the same feature with the other models as well.

### Performance at the OSCE examination

At the OSCE examination, 68 of the fourth-year medical students were assigned to undertake an examination on arthrocentesis skills. 41 of the 68 examinees had attended the hands-on workshop on average one month before the examination, whilst 27 of the examinees hadn’t attended the program. Workshop attendees achieved a significant higher total score than non-attendees (16.2 vs 14.8 points, *p* < 0.05), whilst a tendency towards a better performance among workshop attendees was found in the subsections of the evaluation (see Table 5).

**Table 5:** Performance at the OSCE examination, workshop attendees vs. non-attendees

		Professionalism (0–6)	Clinical skills (0–9)	Reasoning (0–5)	TOTAL (0–20)
**Attendees (*N*** **= 41)**	Mean Min – max	5 3–6	7.1 5–9	4.1 2–5	16.2 12–20
**Non-attendees (*N*** **= 27)**	Mean Min – max	4.7 3–6	6.3 5–9	3.8 2–5	14.8 12–20
**p**		0.22	0.06	0.14	< 0.05

## Discussion

A basic knowledge of musculoskeletal examination and joint injection techniques is essential for medical practitioners. Therefore, improvement in education of musculoskeletal conditions is necessary for doctors as well as for medical students. The results of this study demonstrate that a hands-on arthrocentesis workshop is well suited to enhance both the procedural skills and the self-confidence towards joint injection procedures in fourth year medical students. It also shows that a dedicated “workshop” is achieving better results in terms of skills enhancement than a simple intermittent lecture and teaching as part of other components of a course. Trainees profit from the opportunity to practice their techniques without causing harm to a patient and can repeat the procedure until they feel comfortable. The results of this study are in accordance with previous studies [9–12], that demonstrated improvements in self-confidence and performance of joint and soft tissue injections. Furthermore, this study showed that the workshop might have contributed to the improved OSCE scores in workshop attendees.

Vogelgesang et al. [9] compared residents randomized in three groups and receiving either a lecture on joint injection (group one), a lecture and an injection workshop on models (group two) or bedside instruction if there was an opportunity for a procedure (group three). Whilst medical knowledge was higher in the groups attending the lecture (groups one and two), performance on a practical test with injection models was best with participants assigned to group two. Unlike the findings of Vogelgesang et al., competence on a knee simulator and self-confidence were similar in both the hands-on and the lecture only group in a study published by Leopold et al. [12]. This finding might be due to differences in training level in between the participants of the two studies, as Leopold studied seasoned practitioners, whilst this educational program may better benefit students and residents. A clear short term benefit of the arthrocentesis workshop on synthetic anatomy models was also reported by Barilla-Labarca et al. [10]. However, the question as to whether or not residual benefits are still maintained over time is a matter of debate. In our own series, re-assessment of the skills among the OSCE examination occurred on average one month after the workshop itself. For that reason, although the short-term benefit could be demonstrated with a significant higher total score among workshop attendees, the longevity of this instructional course can’t be derived beyond the year exams. Jolly et al. [2] showed that the mean comfort scores on theoretical and practical aspects of the procedures decreased from immediately after the workshop to a follow up point 10.5 months later, but the scores were still significantly higher than the pre-workshop scores.

Berman et al. [11] evaluated the efficacy of several modalities used for training in arthrocentesis, including the use of cadavers and synthetic joint models. He observed that the use of cadavers permitted trainees to practice arthrocentesis in a more realistic setting. This seemed to facilitate a more rapid establishment of comfort when compared to synthetic anatomic models. However, the advantages of synthetic models, aside from their portability and reusability, include the lower costs as well as the ability to revisit the model for later review at any time. Whilst the tactile qualities of an actual procedure may be limited when using synthetic models in comparison to the cadavers, future improvements in design and technical features in simulation joint models may lower the disparities.

The synthetic models used in this series were rated as good by the participants, although there were some differences noted. Students reported that they preferred the aspiration models to the electronic models as they had the opportunity of physically seeing success with the fluid being drawn out. This provided a realistic scenario, and most of the students wished the feature to be available with the other models. In our workshop, only the knee and spine models allowed for fluid aspiration upon correct placement of the needle. This may also have had an impact on how rapidly confidence with the procedure was achieved. Possibly, this was one of the reasons why participants felt most comfortable to perform an arthrocentesis of the knee at the end of the workshop, although many reasons could explain this finding. The knee is a big and easily accessible joint. Performance of an arthrocentesis at the knee is among the most common arthrocentesis procedures, and different approaches allow safe performance. It doesn’t explain though why the students felt the least confident to perform spinal aspiration procedures at the end of the workshop. A possible explanation could be that the spine model was the oldest among the models and aspiration of fluid was more difficult than with the knee model**.** Further studies exploring the designs of simulated models would be useful to determine if better models can be developed. This could be particularly relevant with the opportunity of departmental 3D printers allowing replacement parts to be made easily available.

Leopold et al. [12] as well as Barilla-Labarca et al. [10] stated that higher pretest self-reported confidence was frequently inversely related to competence. The latter suggested that self-assessment questionnaires can be unreliable and may misinterpret confidence for competence, however this was not indicated in this study. In contrast, the self-assessment matched the results of the medical knowledge questionnaire, suggesting that the students had a good perception of their own capabilities.

### Limitations

This study has a number of limitations. Firstly, the study had only a short-term follow-up to explore sustainability of the knowledge and skills from the program. The enhancement of personal skills was clearly demonstrated, but the long-term benefit on skills and self-confidence during the participants’ real-world practice remains unknown. Acquired comfort with knowledge and practical aspects of joint procedures may undergo attrition over time. Further long-term prospective studies using validated assessment tools would be ideal to address this concern and to prove, whether a longer lasting beneficial effect on the learners’ skills can be achieved.

Secondly, the relatively small number of participants and the lack of demographic data are limiting factors that might have influenced the results in various ways. When rating the workshop, the students may not have felt comfortable to provide negative feedback or to give a bad rating in case identification was possible. This was despite the fact that the impact survey was completed anonymously. Thus, an overrating of the workshop could have occurred. Furthermore, all students had access to additional learning material aside from the workshop, which could be considered as confounding variables. The group might not be representative for all medical students in their fourth year, so that it remains uncertain whether the results of the study would be reproduced in another group. However, given that the structure of the workshop was not complicated to undertake, it would be possible to set up a standardized course to other medical students and medical schools. In contrast, the limited number of participants could also be seen as a strength, because it allowed every student to practice on the models as much as desired, with only 2.5 students sharing one model. As for the OSCE examination, the small number of participants certainly influenced the power of the results. Potentially, a higher volume would have shown significantly better performance in the subsections of the evaluation instead of just in the overall score, but further studies need to be completed in order to comment on this assumption.

The number of available models and the different technical features of the individual models could be another limiting factor to this study. Whilst there were four knee models available for twenty students, the number of models of the spine (two), elbow and shoulder (one each) were lower. The chance to practice on some of the models was therefore potentially limited and could have had an impact on the student’s confidence with the procedure. However, the aim of the study was not to compare the suitability of different injection models, and there are other factors that explain the differing confidence to the procedure on different locations as stated above.

With any self-reported questionnaire social desirability bias is present and is a limitation. However, we proactively attempted to limit this by having some unstructured questions and stressing to the students that the answers were anonymised and purely used to provide feedback on ways we can improve the course in future. Furthermore, we did a second proxy questionnaire at the end of the MSK module asking students about many elements of the course with many forced choice questions where students highlighted the joint injection workshops as one of the highlights. Although this will have not completely mitigated social desirability bias we followed Nederhof*s* [13] methods to reduce this affect.

## Conclusions

A single session, half-day hands-on workshop for arthrocentesis procedures is well suited to increase procedural skills and self-confidence towards the procedure in fourth year medical students. The participants developed important baseline knowledge necessary to perform arthrocentesis procedures safely. Workshops for medical students, in conjunction with other learning activities, not only help learners to gain confidence and to practice invasive techniques without risk to a patient, they allow students to gain knowledge, comprehension and application with time for focused learning in a more structured way, possibly enabling students to progress up Bloom’s taxonomy faster and more efficiently.

## Supplementary information

**Additional file 1.** Practical skills development form (Knee)

**Additional file 2. ** Self-assessment questionnaire

**Additional file 3. ** Medical knowledge check

**Additional file 4.** Impact survey

## Data Availability

Data and materials are available upon request from the University of Adelaide.

## Abbreviations

OSCE: Objective structured clinical examination
MBBS: Bachelor of medicine / bachelor of surgery
MSK: Musculoskeletal
AC joint: Acromioclavicular joint
