# Supplementary material for: The impact of a hands-on arthrocentesis workshop in undergraduate medical education
Source: BMC Med Educ. 2020 Aug 10;20:260. doi: 10.1186/s12909-020-02174-6 (PMC7419181; doi:10.1186/s12909-020-02174-6)
Supplement: Supplementary file 1 — Additional file 1. Practical skills development form (Knee) [file 12909_2020_2174_MOESM1_ESM.docx]

**Appendix 1: Practical skills development form (Knee)**

**Practical skills development**

Practise your procedural skills on the models provided and adhere to the following work flow. Tick when successfully accomplished.

**KNEE**

| Time out and consent | - Obtain consent (reason, risks, benefits, alternatives) - Check identity - Correct procedure (aspiration or injection, medication for injection) - Correct side |
| --- | --- |
| Position patient | - Supine, knee extended or slightly flexed up to 20°) - Exposure area of need |
| Check equipment | - Syringe size - Needle size - Separate drawing up needle - Consumables in date - Desinfectant - Area towel - Swab |
| Identify landmarks | - Patella (medial and lateral edge) - Patellar tendon - Quadriceps tendon - Femoral condyles - Femoral epicondyles - Joint line - Tibial tuberosity |
| Mark approach | - Superolateral approach - Superomedial approach |
| Skin preparation | - Removal of extensive hair - Desinfection - Application of area towel |
| Performance of procedure | - Draw up medication - Insert needle into joint - Aspirate - Inject medication - Retract needle - Apply dressings |
| (Evaluation of aspirate) | - Volume - Clarity - Color - Viscosity - White blood cell count - Polymorphonuclear cells (%) - Culture - Crystals |
| Patient education | - Analgesia - Wound care - Activity and Limitations - Follow up - Red flags |
