# Supplementary material for: The impact of a hands-on arthrocentesis workshop in undergraduate medical education
Source: BMC Med Educ. 2020 Aug 10;20:260. doi: 10.1186/s12909-020-02174-6 (PMC7419181; doi:10.1186/s12909-020-02174-6)
Supplement: Supplementary file 2 — Additional file 2. Self-assessment questionnaire [file 12909_2020_2174_MOESM2_ESM.docx]

**Appendix 2: Self-assessment questionnaire**

**Self-assessment questionnaire**

Please rate your level of confidence (*encircle the most appropriate number*) in your knowledge of …

1. Performance of arthrocentesis in general

| **Not confident at all** | |  |  |  |  |  |  |  |  | **Very confident** | |
| --- | --- | --- | --- | --- | --- | --- | --- | --- | --- | --- | --- |
|  | 0 | 1 | 2 | 3 | 4 | 5 | 6 | 7 | 8 | 9 |  |

1. Anatomy of the knee

| **Not confident at all** | |  |  |  |  |  |  |  |  | **Very confident** | |
| --- | --- | --- | --- | --- | --- | --- | --- | --- | --- | --- | --- |
|  | 0 | 1 | 2 | 3 | 4 | 5 | 6 | 7 | 8 | 9 |  |

1. Performance of arthrocentesis – knee

| **Not confident at all** | |  |  |  |  |  |  |  |  | **Very confident** | |
| --- | --- | --- | --- | --- | --- | --- | --- | --- | --- | --- | --- |
|  | 0 | 1 | 2 | 3 | 4 | 5 | 6 | 7 | 8 | 9 |  |

1. Anatomy of the shoulder

| **Not confident at all** | |  |  |  |  |  |  |  |  | **Very confident** | |
| --- | --- | --- | --- | --- | --- | --- | --- | --- | --- | --- | --- |
|  | 0 | 1 | 2 | 3 | 4 | 5 | 6 | 7 | 8 | 9 |  |

1. Performance of arthrocentesis – glenohumeral joint

| **Not confident at all** | |  |  |  |  |  |  |  |  | **Very confident** | |
| --- | --- | --- | --- | --- | --- | --- | --- | --- | --- | --- | --- |
|  | 0 | 1 | 2 | 3 | 4 | 5 | 6 | 7 | 8 | 9 |  |

1. Performance of arthrocentesis – subacromial space

| **Not confident at all** | |  |  |  |  |  |  |  |  | **Very confident** | |
| --- | --- | --- | --- | --- | --- | --- | --- | --- | --- | --- | --- |
|  | 0 | 1 | 2 | 3 | 4 | 5 | 6 | 7 | 8 | 9 |  |

1. Performance of arthrocentesis – acromioclavicular-joint

| **Not confident at all** | |  |  |  |  |  |  |  |  | **Very confident** | |
| --- | --- | --- | --- | --- | --- | --- | --- | --- | --- | --- | --- |
|  | 0 | 1 | 2 | 3 | 4 | 5 | 6 | 7 | 8 | 9 |  |

1. Anatomy of the elbow

| **Not confident at all** | |  |  |  |  |  |  |  |  | **Very confident** | |
| --- | --- | --- | --- | --- | --- | --- | --- | --- | --- | --- | --- |
|  | 0 | 1 | 2 | 3 | 4 | 5 | 6 | 7 | 8 | 9 |  |

1. Performance of arthrocentesis - elbow

| **Not confident at all** | |  |  |  |  |  |  |  |  | **Very confident** | |
| --- | --- | --- | --- | --- | --- | --- | --- | --- | --- | --- | --- |
|  | 0 | 1 | 2 | 3 | 4 | 5 | 6 | 7 | 8 | 9 |  |

1. Anatomy of the spine

| **Not confident at all** | |  |  |  |  |  |  |  |  | **Very confident** | |
| --- | --- | --- | --- | --- | --- | --- | --- | --- | --- | --- | --- |
|  | 0 | 1 | 2 | 3 | 4 | 5 | 6 | 7 | 8 | 9 |  |

1. Performance of arthrocentesis - spine

| **Not confident at all** | |  |  |  |  |  |  |  |  | **Very confident** | |
| --- | --- | --- | --- | --- | --- | --- | --- | --- | --- | --- | --- |
|  | 0 | 1 | 2 | 3 | 4 | 5 | 6 | 7 | 8 | 9 |  |

1. Obtaining consent for the procedure

| **Not confident at all** | |  |  |  |  |  |  |  |  | **Very confident** | |
| --- | --- | --- | --- | --- | --- | --- | --- | --- | --- | --- | --- |
|  | 0 | 1 | 2 | 3 | 4 | 5 | 6 | 7 | 8 | 9 |  |

1. Risks of arthrocentesis

| **Not confident at all** | |  |  |  |  |  |  |  |  | **Very confident** | |
| --- | --- | --- | --- | --- | --- | --- | --- | --- | --- | --- | --- |
|  | 0 | 1 | 2 | 3 | 4 | 5 | 6 | 7 | 8 | 9 |  |

1. Indications for steroid injection

| **Not confident at all** | |  |  |  |  |  |  |  |  | **Very confident** | |
| --- | --- | --- | --- | --- | --- | --- | --- | --- | --- | --- | --- |
|  | 0 | 1 | 2 | 3 | 4 | 5 | 6 | 7 | 8 | 9 |  |

1. Contraindications for steroid injections

| **Not confident at all** | |  |  |  |  |  |  |  |  | **Very confident** | |
| --- | --- | --- | --- | --- | --- | --- | --- | --- | --- | --- | --- |
|  | 0 | 1 | 2 | 3 | 4 | 5 | 6 | 7 | 8 | 9 |  |

1. Choice of corticosteroid for injection

| **Not confident at all** | |  |  |  |  |  |  |  |  | **Very confident** | |
| --- | --- | --- | --- | --- | --- | --- | --- | --- | --- | --- | --- |
|  | 0 | 1 | 2 | 3 | 4 | 5 | 6 | 7 | 8 | 9 |  |

1. Quantity of corticosteroid for injection

| **Not confident at all** | |  |  |  |  |  |  |  |  | **Very confident** | |
| --- | --- | --- | --- | --- | --- | --- | --- | --- | --- | --- | --- |
|  | 0 | 1 | 2 | 3 | 4 | 5 | 6 | 7 | 8 | 9 |  |

1. Frequency of injection

| **Not confident at all** | |  |  |  |  |  |  |  |  | **Very confident** | |
| --- | --- | --- | --- | --- | --- | --- | --- | --- | --- | --- | --- |
|  | 0 | 1 | 2 | 3 | 4 | 5 | 6 | 7 | 8 | 9 |  |

1. Post-injection advice

| **Not confident at all** | |  |  |  |  |  |  |  |  | **Very confident** | |
| --- | --- | --- | --- | --- | --- | --- | --- | --- | --- | --- | --- |
|  | 0 | 1 | 2 | 3 | 4 | 5 | 6 | 7 | 8 | 9 |  |
