# Supplementary material for: The impact of a hands-on arthrocentesis workshop in undergraduate medical education
Source: BMC Med Educ. 2020 Aug 10;20:260. doi: 10.1186/s12909-020-02174-6 (PMC7419181; doi:10.1186/s12909-020-02174-6)
Supplement: Supplementary file 3 — Additional file 3. Medical knowledge check [file 12909_2020_2174_MOESM3_ESM.docx]

**Appendix 3: Medical knowledge check**

**Medical knowledge check**

**Anatomy:**


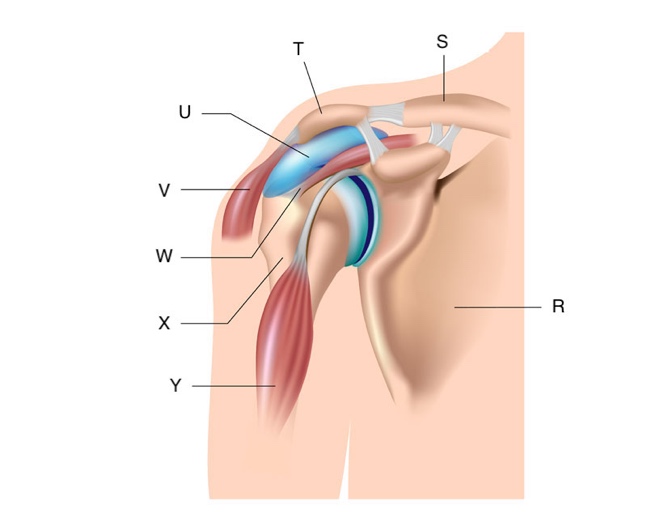

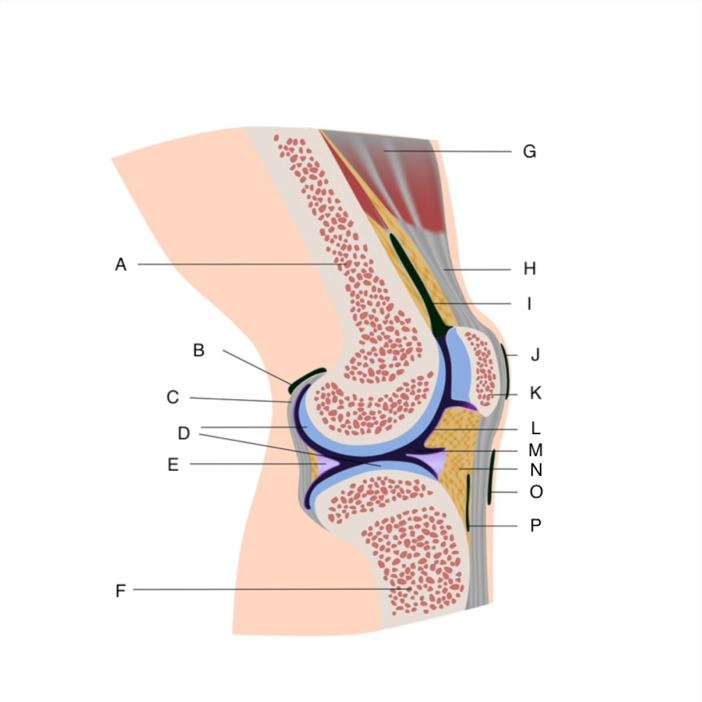


Please assign one letter to each anatomical structure given in the list below and write the letter into the according line. Not every single letter will be assigned as there are more letters offered than anatomical structures.

| **Letter assigned** | **Anatomical structure** |  | **Letter assigned** | **Anatomical structure** |
| --- | --- | --- | --- | --- |
|  | Acromion |  |  | Meniscus |
|  | Anterior cruciate ligament |  |  | Medial collateral ligament |
|  | Articular cartilage |  |  | Patella |
|  | Biceps muscle |  |  | Posterior cruciate ligament |
|  | Clavicle |  |  | Prepatellar bursa |
|  | Coracoid process |  |  | Quadriceps muscle |
|  | Femur |  |  | Subscapular fossa |
|  | Fibula |  |  | Subacromial bursa |
|  | Humerus |  |  | Suprapatellar recess |
|  | Hoffa fatpad |  |  | Tibia |
|  | Lateral collateral ligament |  |  |  |

**Diagnostics - Synovial Fluid Analysis**

A 65 year old man presents to the Emergency Room with a painful, swollen right knee. His symptoms started last night and he remembers no precipitating event. Nothing like this has ever happened to him before. On examination, he has a temperature of 38°C and has a swollen, warm and tender right knee. The knee range of motion is markedly limited and very painful. You decide to aspirate the knee. Indicate which of the following laboratory studies should be done on this patient's synovial fluid by marking each item true or false.

| **True** | **False** |  | **Examination** |
| --- | --- | --- | --- |
| □ | □ |  | Cell Count with differential |
| □ | □ |  | Crystal examination |
| □ | □ |  | Gram stain |
| □ | □ |  | Glucose |
| □ | □ |  | Lactate |
| □ | □ |  | LDH |
| □ | □ |  | Ph |
| □ | □ |  | Protein |
| □ | □ |  | Synovial Fluid Culture |

**Corticosteroid Injections – Preparations**

1. Betamethasone Sodium Phosphate/Betamethasone Acetate
2. Methylprednisolone Acetate
3. Triamcinolone Acetonide
4. Triamcinolone Hexacetonide

For each statement below, select the most appropriate corticosteroid preparation from the list above. Write the most appropriate letter into the box provided. Use each answer only once.

| **Letter** | **Preparation** |
| --- | --- |
|  | Nonfluorinated corticosteroid preparation (better for soft‐tissue injection) |
|  | Least water soluble corticosteroid preparation (better for intra‐articular injection) |
|  | A *more* water soluble corticosteroid preparation (better for soft‐tissue injection) |

**Corticosteroid Injections - Contraindications (relative) and adverse effects of intra‐articular corticosteroid injections**

1. Atypical articular anatomy
2. Bacteremia
3. Cataracts
4. Cellulitis overlying the joint
5. Cushingoid facies
6. Cutaneous atrophy
7. Cutaneous pigmentation changes
8. Fractured joint
9. Hypercalcemia
10. Hypertension
11. Joint infection
12. Post‐injection flare
13. Renal tubular acidosis
14. Striae

From the list above, select 4 common (**relative) contraindications** to giving an intra‐articular corticosteroid injection and write the according letters into the boxes.

|  |  |  |  |
| --- | --- | --- | --- |

From the list above, select 4 well‐described (although uncommon) **adverse effects** of giving a single intraarticular corticosteroid injection and write the according letters into the boxes.

|  |  |  |  |
| --- | --- | --- | --- |

**Infections**

The approximate rate of infection that is published as a complication of arthrocentesis is *(tick the box with the most appropriate answer)*:

| - 0.2 (2 out of 10 injections) |  |
| --- | --- |
| - 0.02 (2 out of 100 injections) |  |
| - 0.002 (2 out of 1,000 injections) |  |
| - 0.0002 (2 out of 10,000 injections) |  |
| - 0.00002 (2 out of 100,000 injections) |  |
| - 0.000002 (2 out of 1,000,000 injections) |  |
