# Supplementary material for: The impact of a hands-on arthrocentesis workshop in undergraduate medical education
Source: BMC Med Educ. 2020 Aug 10;20:260. doi: 10.1186/s12909-020-02174-6 (PMC7419181; doi:10.1186/s12909-020-02174-6)
Supplement: Supplementary file 4 — Additional file 4. Impact survey [file 12909_2020_2174_MOESM4_ESM.docx]

**Appendix 4: Impact survey**

**Impact survey**

*Please answer the questions by ticking the box with the most appropriate answer*

|  |  |  |  | Yes | No |
| --- | --- | --- | --- | --- | --- |
| **1.** | Do you think, this workshop is useful to enhance the skills in arthrocentesis technique | | | □ | □ |
| **2.** | Do you think, this workshop influenced your future practice habits? | | | □ | □ |
| **3.** | Do you think, every doctor should complete a workshop like this first before performing an injection on themselves? | | | □ | □ |
| **4.** | Would you recommend this workshop to other students? | | | □ | □ |
| **5.** | Do you think you feel comfortable to perform an arthrocentesis on your own after completing this workshop? If so, how many attempts did it take until you felt comfortable with the procedure? | | |  |  |
|  |  | Comfortable? | | Attempts | |
|  |  | Yes | No | *(number)* | |
|  | - Overall | □ | □ |  | |
|  | - Knee | □ | □ |  | |
|  | - Glenohumeral joint | □ | □ |  | |
|  | - Subacromial space | □ | □ |  | |
|  | - Acromioclavicular joint | □ | □ |  | |
|  | - Elbow | □ | □ |  | |
|  | - Spine | □ | □ |  | |

*Please answer the questions by encircling the most appropriate number*

1. How would you rate the quality of the equipment provided?

| **Poor** | |  |  |  |  |  |  |  |  | **Very good** | |
| --- | --- | --- | --- | --- | --- | --- | --- | --- | --- | --- | --- |
|  | 0 | 1 | 2 | 3 | 4 | 5 | 6 | 7 | 8 | 9 |  |

1. How would you rate the quality of the didactic instruction?

| **Poor** | |  |  |  |  |  |  |  |  | **Very good** | |
| --- | --- | --- | --- | --- | --- | --- | --- | --- | --- | --- | --- |
|  | 0 | 1 | 2 | 3 | 4 | 5 | 6 | 7 | 8 | 9 |  |

1. How would you rate the quality of the workshop overall?

| **Poor** | |  |  |  |  |  |  |  |  | **Very good** | |
| --- | --- | --- | --- | --- | --- | --- | --- | --- | --- | --- | --- |
|  | 0 | 1 | 2 | 3 | 4 | 5 | 6 | 7 | 8 | 9 |  |

*Please share your thoughts with us and write your comments into the lines below*

|  | What was particularly positive? *(please provide at least 3 positive findings)* | | | |
| --- | --- | --- | --- | --- |
|  |  |  |  |  |
|  |  |  |  |  |
|  |  |  |  |  |
|  |  |  |  |  |
| **10.** | What was negative? *(please provide at least 3 negative findings)* | | | |
|  |  |  |  |  |
|  |  |  |  |  |
|  |  |  |  |  |
|  |  |  |  |  |
| **11.** | How can we improve? *(Please provide at least 1 suggestion for improvements)* | | | |
|  |  |  |  |  |
|  |  |  |  |  |
|  |  |  |  |  |
|  |  |  |  |  |
